# Translation, cultural adaptation, and psychometric properties of the *MISSCARE survey OR* - *Swedish version*

**DOI:** 10.1186/s12912-025-03548-1

**Published:** 2025-07-05

**Authors:** Ann-Christin von Vogelsang, Karin Falk-Brynhildsen, Sofia Erestam, Christine Leo Swenne, Eva Torbjörnsson

**Affiliations:** 1 Department of Neurobiology, Care Sciences and Society, Karolinska Institutet, Stockholm, Sweden; 2 Department of Neurosurgery, Karolinska University Hospital, Stockholm, Sweden; 3 Faculty of Medicine and Health, School of Health Sciences, Örebro University, Örebro, Sweden; 4 Institute of Health and Care Science, University of Gothenburg, Gothenburg, Sweden; 5 Department of Public Health and Caring Sciences, Uppsala University, Uppsala, Sweden; 6 Department of Clinical Science and Education Södersjukhuset, Karolinska Institutet, Stockholm, Sweden; 7 Department of Perioperative and Intensive Care, Karolinska Institutet, Stockholm, Sweden

**Keywords:** Missed nursing care, Patient safety, Perioperative nursing, Psychometrics

## Abstract

**Background:**

Missing nursing care in the perioperative context affects quality of care and patient safety. More validated language versions of *the MISSCARE Survey OR* enable local, national, and international comparisons. The aim was to translate and culturally adapt the American *MISSCARE Survey OR* to the Swedish perioperative context and assess the instrument’s psychometric properties.

**Methods:**

A cross-sectional validation study with translation and cultural adaptation was conducted in eight steps according to the International Society for Pharmacoeconomics and Outcomes Research guidelines. To assess the psychometric properties, data were collected via a web survey distributed at a Swedish national conference for operating room nurses. The data collection was concluded in November 2023. Acceptability, construct validity and internal consistency were analyzed.

**Results:**

Cultural adaptation resulted in the omission of two items from the section measuring missed elements of care. A total of 107 (out of 261 invited operating room nurses) respondents were included in the psychometric testing, leading to a response rate of 41%. Acceptability was high, and 95.3% of the respondents answered all the items. The factor analysis on missed elements of care was inconclusive and was therefore regarded as a list of nursing actions. With respect to reasons for missed nursing care, the factor analysis resulted in a three-factor solution, which explained a cumulative variance of 62.6%. The alpha coefficients for internal consistency for the three factors ranged from 0.760 to 0.947.

**Conclusions:**

The Swedish *MISSCARE Survey OR* is a valid instrument with sufficient psychometric properties and provides a reliable measure of missed nursing care and its reasons. Usage could be valuable for nursing managers and directors, who are responsible for care processes and strategic care planning.

## Background

Several concepts have been developed to describe required nursing care that is omitted – either partially, entirely, or delayed. These concepts often overlap, and a unified global conceptualization remains lacking [1]. Commonly used terms, which are frequently employed interchangeably, include missed nursing care (MNC), omitted care [2], unfinished nursing care [3, 4], care left undone [5], and care rationed or missed [1]. However, some authors have argued that the concept of rationing of care is related yet conceptually distinct, as it refers to a deliberate and reasoned decision to withhold care [6]. MNC is a global concern, which was highlighted when Griffiths et al. [7], in a systematic review, reported that overall 88% of nurses across 12 countries working in inpatient hospital wards reported that some elements of care were missed during their last shift. This is serious in light of the consequences of MNC. In general acute hospitals, MNC is associated with patient death after common surgical procedures [8]. A systematic review examined the impact of MNC on patient outcomes and revealed that MNC is associated with several adverse events and negative patient outcomes, such as medication errors, bloodstream infections, pneumonia, urinary tract infections, pressure ulcers and increased hospital readmissions. Moreover, MNC also has been associated with lower reported patient and informal caregiver experiences and satisfaction ratings [9]. A recent review including 24 studies revealed that several studies reported a significant correlation between higher levels of MNC and an increased likelihood of nurses reporting an intention to leave their job. Several factors influence the relationship between MNC and the intention to leave, either as a driving factor, a predictor, or a mediator: i.e., workload, burnout, organizational support, and job satisfaction [10]. In a systematic review, the antecedents of missed nursing care (MNC) were categorized into three levels: unit-level factors (e.g., workload, work environment, staffing levels), nurse-level factors (e.g., age, gender, education, professional experience), and patient-level factors (e.g., clinical instability) [11].

There are several surveys used to measure MNC, with the *MISSCARE Survey*, constructed by Kalisch & Williams [2], being the most widely used and validated tool. It has been used worldwide for more than a decade in various in-hospital settings and contexts: medical, surgical, intermediate and intensive care units; geriatric and rehabilitation units; and community care [12]. However, the original version of the *MISSCARE Survey* is not appropriate for use in the perioperative context. Perioperative care is typically divided into three distinct phases. The *preoperative phase* begins when the decision for surgical intervention is made and concludes with the patient’s transfer to the operating room (OR). The *intraoperative phase* starts upon the patient’s arrival in the OR and ends with their transfer to the post-anesthesia care unit (PACU). Finally, the *postoperative phase* commences with admission to the PACU and continues until the resolution of surgical sequelae [13].

New instruments to measure MNC have been developed for the care provided in PACUs [14, 15] and the instruments were inspired by the work of Kalisch & Williams [2]. However, the nursing care provided during the intraoperative phase differs significantly from all the previously mentioned contexts and warranted the development of an MNC survey suited to OR nursing care [16]. The OR suite is among the most restricted and inaccessible areas within a hospital. Entry is limited to authorized personnel and patients, all of whom are required to wear surgical attire [17]. The OR environment is characterized by a high degree of technological complexity. Patients undergoing surgical procedures are typically rendered fully dependent on healthcare providers due to the effects of general anesthesia, which inhibits movement and the perception of pain [18]. Within the OR, nurses are responsible for preparing both the patient and the surgical environment, as well as facilitating communication among members of the surgical team. In contrast, nurses working in medical-surgical units are generally tasked with interventions such as ambulation, bathing, and mouth care—activities that are not typically included in the scope of practice for OR nurses [14]. The construction process of an instrument started in 2011 [19] and thereafter resulted in the *MISSCARE Survey OR* [16, 20].

There are other patient safety risks since nursing care in the OR differs significantly from inpatient care in medical-surgical units, intensive care units or PACUs [19]. It has been estimated that of all adverse events within a hospital, 50% occur during surgery [21]. Intraoperative adverse events include, for example, wrong-patient or wrong-site surgery, communication errors, medication errors, and unintended retained foreign objects after surgery [22–24]. A near-miss event in healthcare is defined as an event that did not happen but would have caused serious harm if it did. A recent mixed-method review of near-miss events in the OR revealed that a lack of information sharing, communication and disruptions are contributing factors for near-miss events to occur [25]. In a study by Cohoon [26], the surgical team contributed to near-miss events, and inconsistent information and incorrect monitoring were the predominant causal factors. In the development of the *MISSCARE Survey OR*, all these safety risks were scrutinized, along with World Health Organization guidelines for safe surgery, including the use of the Safe Surgery Checklist [27]. The differences between the elements of nursing care included in the *MISSCARE Survey OR* version in comparison to the *MISSCARE Survey* used in other contexts were depicted.

The original *MISSCARE Survey OR* consists of three sections: a background data section, a section with 32 items on missed elements of perioperative nursing care (section A), where the respondents answer on a six-point Likert-type scale (‘*never missed’*,* ‘rarely missed’*, ‘*occasionally missed’*, *‘frequently missed’*, ‘*always missed’*, and an *‘not applicable’* option).

Thereafter follows a section with 17 items on reasons for MNC (section B), where the respondents rate reasons for MNC on a four-point Likert-type scale *(‘not a reason’*,* ‘minor reason’*,* ‘moderate reason’ and ‘significant reason’*) [16]. *The MISSCARE Survey OR* underwent psychometric evaluation in 2021, involving participation from over 1,600 operating room nurses (ORNs) across the United States. Exploratory factor analysis of part A identified five distinct factors (Legal requirements, Preparation, Safety, Communication, and Closing routine) which collectively accounted for 44% of the total variance. In part B, a four-factor solution (Urgency, Staffing, Materials, and Teamwork) explained 53% of the variance. The instrument demonstrated high acceptability. Cronbach’s alpha coefficients indicating satisfactory internal consistency, ranging from 0.71 to 0.84 for Part A and 0.74 to 0.90 for Part B [16].

The most frequently reported reasons for MNC are similar worldwide. A recent systematic literature review with meta-analysis revealed that the most frequently reported reasons were *‘unexpected rise in patient volume and/or acuity on the unit’*, *‘inadequate number of staff’*, and *‘urgent patient situation’* [28]. Equivalent items are included in the *MISSCARE Survey OR* version but differ to some extent from the *MISSCARE Survey*, and include specific reasons only applicable in intraoperative care, such as *‘unexpected change in planned surgical procedure’* and *‘inadequate number of unlicensed assistive personnel to help open cases and clean rooms’* [16].

The *MISSCARE Survey OR*, after being constructed in the U.S. perioperative context, has been used in Australia and Egypt. When used in Australia, no descriptions of cultural adaptations or further validations have been provided [29, 30]. When used in Egypt [31], translation to Arabic, cultural adaptation to Egyptian culture and psychometric testing were described for the section on missed elements of care but not for the section evaluating reasons for MNC. In the Egyptian version, one answer option was omitted (not applicable), and a five-point Likert-type scale (‘*never missed’*,* ‘rarely missed’*, ‘*occasionally missed’*, *‘frequently missed’*, and ‘*always missed’*) was used, contrary to the original *MISSCARE Survey OR* version, which uses a six-point Likert-type scale (‘*never missed’*,* ‘rarely missed’*, ‘*occasionally missed’*, *‘frequently missed’*, ‘*always missed’*, and an *‘not applicable’* option).

To date, published studies on MNC in the OR context differ in the inclusion criteria of samples. The samples used in the Australian version by Gillespie et al. [29, 30] included nurses who practiced in perioperative clinical roles and included circulating, instrument, anesthetic, and PACU nurses and educators; whereas the samples used in the U.S. *MISSCARE Survey OR* validation study [16], the study by Marsh [20], and the Egyptian study [31] ORNs. Although nursing roles in the OR are similar worldwide, there may be variations in how the tasks within the ORs are operationalized [32]. Worldwide, the most common roles are a scrub person that dons sterile attire and works in tandem with a circulator who works outside the sterile field. By working in tandem, the scrub person and the circulator prepare the instruments and supplies for the surgical procedure, safeguard the sterile field, manage specimens, anticipate surgical team needs and manage other indispensable intraoperative tasks [19]. However, for the different nursing roles in the OR, there are variations in educational training and certification requirements across countries [32]. In the U.S., the scrub person may be a registered nurse, a surgical technologist, or a licensed practical nurse [33]. The scrub person in Sweden is a certified ORN, and the circulation role is most often performed by a nurse assistant [34]. The professional title of ORN is protected by law in Sweden and may be used only by nurses registered with the Swedish National Board of Health and Welfare who hold a bachelor’s degree and who also have a postgraduate diploma (60 credits) in OR nursing, which results in both a professional title and a master’s degree [35].

The *MISSCARE Survey OR* is a reliable, valid indicator of the extent of and reasons for MNC and is used in American, Australian, and Egyptian perioperative contexts. However, those contexts differ from Swedish conditions, and the role of an ORN in the U.S., Australia and Egypt is different from the role in Sweden. As a result, we recognize the necessity of adapting and validating the instrument to fit the Swedish healthcare context.

The aim of this study was to translate and culturally adapt the *MISSCARE Survey OR* to the Swedish perioperative context and to assess the instrument’s psychometric properties.

## Methods

### Design

This validation study used a cross-sectional design.

### Samples

Two samples were used in this study. First, a convenience sample of 10 ORNs was used for cognitive interviews in the translation and cultural adaptation process. The inclusion criterion was being a clinically active ORN. Differences in healthcare regions, surgical services, professional experiences, age, and gender were strived for. Second, a national sample of ORNs participating at a profession-specific conference was used. The inclusion criterion was being a clinically active ORN.

### The translation and cultural adaptation process

All five members of the research group are certified ORNs and PhDs, three of whom also are associate professors. All have experience in psychometric testing of instruments. The members have extensive clinical experience in OR nursing within different surgical contexts, such as neurosurgery, general surgery, thoracic surgery, and orthopedic surgery.

The instrument developer, Professor Kalisch, was contacted by the first author for permission to translate and culturally adapt the instrument to Swedish, and permission was granted in August 2022. All available versions of the instrument were scrutinized. The translation and cultural adaptation process was conducted via the method recommended by the International Society for Pharmacoeconomics and Outcomes Research (ISPOR), including forward translation, reconciliation, back translation, harmonization, cognitive debriefing interviews, finalization, and proof reading [36]. The initial forward translation was conducted within the research group, which all are knowledgeable about English-speaking culture but are native Swedish speakers. The first author translated an initial version of the instrument, and the reconciliation process was carried out until consensus was reached within the research group. The back translation was made by consulting a native English-speaking clinically active ORN in Sweden. The first author reviewed the back translation, and slight changes to the instrument were made, followed by a harmonization meeting with the 5-member research group according to recommendations by Wild et al. [36]. During the harmonization meeting, the first author presented items that were identified as conceptually problematic during the translation process. Alternative translation solutions were shared with the group and discussed until consensus was reached. In the next step, cognitive debriefing and interviews were conducted. All the cognitive debriefing results were reviewed by the first author, and slight changes were made using consensus with the research group. The final proofreading was conducted by the first and last authors of this publication.

### Data collection

Debriefing Interviews. The cognitive debriefing interviews were individual and were performed via a study-specific protocol, where all five members in the research group conducted two interviews each (sample of 10). The rationale for conducting these interviews was to evaluate the level of comprehensibility, test alternative translations, and identify any additional issues that might cause confusion [36]. During the interviews, the items were read one by one, probing:

- Are the question and following answer options understandable?
- Could something in the question or answer option be misunderstood?
- Are there suggestions for other wordings of the question?

Psychometric Properties. The data collection to test the psychometric properties was conducted via a web survey at the online survey and reporting platform Webropol [37] during the Swedish Operating Room Nurses Association (SEORNA) annual conference in November 2023. The web survey was reached by scanning a quick-response (QR) code available in the conference program and announcements at the conference venue. The web survey was open for four days. No questions were mandatory for completing the survey.

### Data analysis

Survey data were exported from Webropol into SPSS version 27 (IBM Corp.) Acceptability was evaluated by the number of respondents who completed the scales without omitting any items for both sections A and B [2]. Construct validity was tested through exploratory factor analysis (EFA). A series of EFAs were performed for section A (items related to missed care) and section B (reasons for missed care). EFA was computed via the principal axis factoring method for extraction and orthogonal varimax rotation with Kaiser normalization [38]. An eigenvalue cutoff of 1.0 was used, and the significant factor criterion was 0.35. In the EFA of section A, single median imputation [39] was used for the answer option ‘Not applicable’. Reliability was evaluated by assessing the internal consistency of the items representing the factors, using Cronbach’s alpha.

### Ethical considerations

Ethical approval for this study was deemed unnecessary under the Swedish Act (2003:460) Concerning the Ethical Review of Research Involving Humans [40], as this type of research does not fall within its scope. Only studies involving physical intervention intended to affect research participants, studies posing a risk of harm, studies including biological material, or studies processing sensitive personal data are required to undergo ethical review. None of these conditions applied to this study.

The study was conducted in accordance with good clinical practice and the principles of the Declaration of Helsinki [41]. Written information about the study was provided as an introductory text to the survey, emphasizing its voluntary nature and ensuring confidentiality. By completing the questionnaire, participants provided their informed consent to participate and agreed that their answers could be used for research. Thus, all participants who answered the questionnaire consented participation. The researchers only had access to anonymized data.

## Results

### Characteristics of the samples

For the cognitive interviews in the translation and cultural adaptation process, ten ORNs were used: six women and four men. Their median work experience as an ORN was 10 years (ranging from 1 to 38 years) working in three of Sweden’s six healthcare regions and within the following surgical services: general surgery (*n* = 2), orthopedic surgery (*n* = 2), ear, nose and throat surgery (*n* = 2), thoracic surgery (*n* = 2), vascular surgery (*n* = 1), and neurosurgery (*n* = 1).

A total of 261 ORNs were invited to participate in the cross-sectional validation study, of which *n* = 109 responded. Two surveys were excluded because of incomplete data; thus, 107 ORNs were included, resulting in a response rate of 41%. The demographics are shown in Table 1.

**Table 1:** Characteristics of participants, *n* = 107

Characteristic	*n* (%)
**Age (years)**	
Mean (± SD)	45.8 (± 10.2)
Range	25–74
**Sex**	
Male	7 (6.5)
Female	100 (93.5)
Type of surgical service*	
Ear, nose and throat surgery	44 (41.1)
Eye surgery	3 (2.8)
General surgery	69 (64.5)
Gynecological surgery	58 (54.2)
Hand surgery	33 (30.8)
Neurosurgery	10 (9.3)
Orthopedic surgery	64 (59.8)
Plastic surgery	25 (23.4)
Thoracic surgery	2 (1.9)
Transplantation surgery	17 (15.9)
Trauma Surgery	45 (42.1)
Urological surgery	50 (46.7)
Vascular surgery	31 (29.0)
**Highest academic degree**	
Without academic degree	9 (8.4)
Bachelor	27 (25.2)
Master one-year/two-year	70 (65.4)
Licentiate or PhD	1 (0.9)
**Experience in role**	
≤ 6 months	2 (1.9)
6–24 months	9 (8.4)
2–5 years	17 (15.9)
6–10 years	15 (22.9)
> 10 years	62 (57.9)
Missing	2 (1.9)
**Experience at current unit**	
≤ 6 months	6 (5.6)
6–24 months	19 (17.8)
2–5 years	30 (28.0)
6–10 years	17 (15.9)
> 10 years	35 (32.7)

* The participants were encouraged to answer all types that apply; thus, the percentage exceeds 100%

### Translation and cultural adaptation

In the cultural adaptation process, minor changes in wording were made to the background questions. Two questions were added to the background section of the survey: ‘How do you perceive the quality of care on the ward?’ and ‘How do you perceive patient safety on the ward?’ Both questions were answered on a five-point Likert scale (‘*very good’*,* ‘good’*,* ‘neutral’*,* ‘poor’* and *‘very poor’*).

A time frame was included in the instruction text prior to section A, within the last month. Two items in section A were omitted *(‘Surgical consent is signed*,* dated and witnessed prior to surgery’* and *‘Surgical consent is correct for the procedure’*), since written surgical consent is not used or required prior to surgery in Sweden. For the same reason, the item *‘Surgical team verifies patient*,* consent*,* procedure*,* site*,* and side before incision’* was changed to *‘Surgical team verifies patient*,* procedure*,* site*,* and side before incision’*. The item *‘The team waits 3 minutes for alcohol preps to dry’* was changed according to Swedish guidelines (to wait at least 2 min). In section B, all questions and answer options were retained, and only slight changes in wording were made.

### Psychometric properties

The acceptability of the survey was good; *n* = 102 (95.3%) of the respondents answered all the items in both sections A and B. A total of six items in section A had one missing answer (Table 2), where one respondent failed to respond to four items, which led to an answer rate of 97.2% (*n* = 104). In section B, two items had one missing answer (Table 3), resulting in an answer rate of 98.1% (*n* = 105).

**Table 2:** Missing answers per item and factor analysis part A

Items	Missing answers	Factors and loadings								
		1	2	3	4	5	6	7	8	9
1. Nurse anesthetist and OR nurse controls and verifies the patient’s identity	0						0.646			
2. The patient or patient’s legal guardian (if applicable) verifies the surgical procedure	1			0.598						
3. Surgical site is marked before the anesthesia starts if there is more than one possible location	0								0.640	
4. History and physical is complete and available before surgery ^a)^	0									
5. Abnormal laboratory values are reported to the surgical team	1						0.502			
6. Allergies are documented and reported to the surgical team	0					0.628				
7. Surgical team is notified if patient has a latex allergy	1									0.413
8. Patient’s and legal guardian’s (if applicable) questions regarding surgery have been addressed	1				0.538					
9. Comfort measures are provided to the patient ^b)^	0									
10. Implantable devices are identified and disclosed to the surgical team	0	0.580								
11. Isolation precautions are implemented for patients with infectious diseases ^b)^	0									
12. Needed blood products are ordered and are available before surgery	0	0.382								
13. Preoperative antibiotics are ordered and ready to be administered before surgery	0		0.377			0.445				
14. Sequential compression devices are applied before the start of surgery, if applicable	0									0.708
15. Necessary information has been communicated among team members before transferring the patient to the OR	0				0.556					
16. Patients are transferred to and from the OR bed without harm	0		0.368							
17. Patients are positioned to prevent risk of complications	0	0.393	0.439							
18. Surgical team verifies patient, procedure, site, and side before incision	1	0.635								
19. A single-use dispersive electrode is placed when monopolar electrosurgery is used	0	0.616								
20. The surgical field is monitored for breaks in sterile technique	0	0.406			0.407					
21. Count discrepancies are communicated to the surgeon	0							0.550		
22. Other activities are suspended during the surgical time out	0			0.570						
23. Surgical time out takes place in the OR with the entire surgical team present	0			0.626						
24. Team members’ questions or concerns are resolved before the incision is made	0			0.462		0.449				
25. The alcohol-based antiseptic skin prep solution is allowed to dry for 2 min before draping	1				0.587					
26. A standardized communication tool is used during hand overs	0			0.390						
27. Essential information is communicated at change of staffing: breaks, lunch, shift changes	0					0.359				
28. Surgical counts are performed before hollow organs and/or the wound is closed	0							0.769		
29. Specimens are verified and labeled correctly	0		0.674							
30. Specimens are handled correctly and sent to the laboratory	0		0.783							
Eigenvalues		2.38	2.23	2.17	1.94	1.45	1.35	1.29	1.0	0.94
% of variance		7.94	7.43	7.24	6.48	4.84	4.50	4.30	3.32	3.12

^a)^ Factor loading below 0.30

^b)^ Factor loading below 0.35

Five-factor solution in the original *MISSCARE Survey OR* scale by Kalisch et al. [16]:

*Legal requirements*: items 1–4; *Preparation*: items 5–15; *Safety*: items 16–21;

*Communication*: items 22–27; *Closing routine*: items 28–30

**Table 3:** Missing answers per item, and three factors for the reasons for missed nursing care

Items	Missing answers	Staffing and teamwork	Urgency	Materials
Lack of confidence among surgical team members	0	0.796		
Cooperation or communication problems with nursing personnel in surgical team	0	0.787		
Cooperation or communication problems with surgeon or anesthesia provider	0	0.740		
Lack of flexibility of surgical team members	0	0.652		
Fatigue and/or exhaustion of team members	0	0.640		
Lack of support from management	1	0.614		
Lack of assistance from surgical team members	0	0.600		
Inadequate number or skill mix of staff	0	0.539		
Interruptions during case; pager, phone calls, unnecessary conversations	0	0.519		
Lack of competence skill set for case	0	0.507		
Inadequate number of ancillary staff to help open cases and clean rooms	0	0.429		
Staff complacency (wants to manage by themselves, do not ask for help)	1	0.501		
Unexpected change in planned surgical procedure	0		0.677	
Urgent clinical situations (e.g., a patient’s conditioning worsening)	0		0.676	
Unexpected rise in patient volume and/or acuity	0		0.549	
Supplies, equipment, or instruments not available for surgical procedure	0			0.738
Supplies, equipment, or instruments not functioning properly when needed	0			0.708
Eigenvalues		5.00	2.90	2.75
% of variance		29.39	17.08	16.14

### Section A

A total of 94 surveys were included in the EFA. Single value imputation was used for the ‘Not applicable’ answer option for 13 items, and median values were used. The Kaiser‒Meyer‒Olkin measure was 0.779, indicating that the data were sufficient for EFA. Bartlett’s test of sphericity, χ^2^ (435) = 940.599, *p* < 0.001, revealed that there were patterned relationships between the items. Nine factors were extracted and explained a cumulative variance of 49.16%. However, the factor analysis was considered inconclusive; three of the extracted factors included two items, and one factor comprised solely one item. Three items exhibited factor loadings below the established threshold of 0.35. Four items were cross-loaded, i.e., loaded at 0.32 or higher on two or more factors [33]. The inconclusive EFA for section A is presented in Table 2. According to these results, section A was considered a list of independent nursing actions that were not related to one another. Thus, internal consistency was not calculated.

### Section B

A total of *n* = 105 were included in the EFA. The Kaiser‒Meyer‒Olkin measure was 0.923, indicating that the data were excellent for EFA. Bartlett’s test of sphericity, χ^2^ (136) = 1346,933 *p* < 0.001, revealed a patterned relationship between the items. Three factors were extracted and explained 62.61% of the cumulative variance. The EFA for section B is presented in Table 3. The alpha coefficients for internal consistency of the three factors in section B were *Staffing and teamwork*: 0.947; *Urgency*: 0.760; and *Materials*: 0.807.

## Discussion

This study aimed to translate and culturally adapt the *MISSCARE Survey OR* to the Swedish perioperative context and to assess the instrument’s psychometric properties. The cultural adaptation resulted in the omission of two items from section A. The factor analysis for section A was inconclusive and was therefore regarded as a list of nursing actions. In section B, a three-factor solution was found. The psychometric properties of the *MISSCARE Survey or Swedish versions* were considered sufficient.

The construct validity was tested through a series of EFAs. An EFA facilitates the interpretation of the result, as it is easier to focus on some key factors than different variables [38]. In the original development of the *MISSCARE Survey OR*, Kalisch et al. [16] found a five-factor solution in section A: *Legal requirements*, *Preparation*, *Safety*, *Communication* and *Closing routine* [16], and the same factorization was found in the Egyptian psychometric study by El-Sayed et al. [31]. Although the statistics in our study revealed a patterned relationship between the items in section A, the factor analysis was inconclusive. As a result, section A was a list of independent nursing actions without a necessary connection to one another. One reason for this finding could be that the testing of the original U.S. version [16] and the Egyptian version [31] both included larger sample sizes, which is preferable when using EFA [38]. On the other hand, for the first *MISSCARE Survey*, for use in medical-surgical inpatient wards, the originator Professor Kalisch came to the same conclusion as in section A, i.e., the factor analysis was inconclusive, and section A was regarded as a list of independent nursing actions [2].

The original version of part B contains four factors, whereas the Swedish version consists of only three factors. The difference is that the factors *Teamwork* and *Staffing* are combined into one factor in our version, unlike the original, where they consist of two distinct factors. This may also be a result of our small sample size, since a small sample size may lead to items being misclassified on the wrong factor [42]. The factorization into one factor in our study (*Staffing and teamwork*) implied that 12 items were included, which may contribute to the high alpha coefficient for internal consistency of 0.947, since the alpha value is strongly affected by the length of the scale. A scale may consist of two independent constructs and still have a high alpha value if the scale contains enough items. An alpha value that is too high may indicate redundancy, and an alpha coefficient of a maximum of 0.90 has been recommended [43].

In our EFA, the factor labeled *Materials* only includes two variables. A factor with fewer than three factors is generally unstable [42]. However, the factor analysis in the original U.S. study also has a factor named *Materials*, which includes only two items [16], as does the Egyptian study [31], which are the same items used in our study. Moreover, the alpha coefficient for internal consistency for the factor *Materials* in our study was 0.807, indicating unidimensionality [43].

The response rate of 41% was acceptable considering that response rates in healthcare surveys differ between countries, that web-based surveys have lower response rates than postal surveys do, and that patient surveys have higher response rates than healthcare professional surveys do. The average response rate for web surveys for healthcare professionals is 45.8% (± 25%) [44], which is in line with our response rate.

Having more validated language versions of the *MISSCARE Survey OR* allows for local, national, and international comparisons and contributes to enhancing patient safety by identifying and assessing the risks of adverse events and negative patient outcomes during care processes in the operating room. Using this instrument could be beneficial for nursing managers and directors, who oversee care processes and strategic care planning. Nursing managers’ better understanding of nurses’ concerns, levels of MNC and its reasons can help reduce incidents [45].

### Strengths and limitations of the work

A strength of the study is the high acceptability, i.e., the respondents omitted a few items and indicated that the survey was easy to use. Another strength is the use of a web survey during a conference, which enables fast data collection and the possibility of reaching a nationally targeted population. However, the use of an anonymized web survey that was reached solely by a QR code ruled out the possibility of sending out reminders, which may have contributed to a fairly low response rate.

A major limitation is the small sample size, since larger sample sizes produce more accurate factor solutions. The variables that are subjected to EFA should preferably have five to ten observations [38]. For section B, the subject-to-item ratio was slightly higher than 6:1. However, Costello and Osborne [42] calculated the effects of the subject-to-item ratio when conducting EFAs and reported that the ideal sample size is at least 20:1 for the EFA to produce correct factor solutions. This study validated the Swedish *MISSCARE Survey OR* among ORNs. However, perioperative care also involves other team members, such as the anesthetic nurse and the nurse assistant. Therefore, we recommend including these professions in further testing of the instrument.

## Conclusions

The Swedish *MISSCARE Survey OR* has sufficient psychometric properties and is a valid instrument for assessing MNC in the Swedish perioperative context. Measuring elements of missed care and its reasons contributes to enhancing patient safety by identifying and assessing the risks of adverse events and negative patient outcomes during care processes in the operating room. Usage could be valuable for nursing managers and directors, who are responsible for care processes and strategic care planning.

## Data Availability

The dataset generated and analyzed during the current study is not publicly available because the participants of this study were not informed of or gave consent for their data to be shared publicly. The data are available from the corresponding author upon reasonable request.

## Abbreviations

EFA: Exploratory Factor Analysis
ISPOR: International Society for Pharmacoeconomics and Outcomes Research
MNC: Missed Nursing Care
OR: Operating room
ORN: Operating room nurse
PACU: Post Anesthesia Care Unit
SEORNA: Swedish Operating Room Nurses Association
